# Rabbit intakes and predictors of their length of stay in animal shelters in British Columbia, Canada

**DOI:** 10.1371/journal.pone.0300633

**Published:** 2024-04-24

**Authors:** Ashley Sum Yin U., Cheng Yu Hou, Alexandra Protopopova

**Affiliations:** Animal Welfare Program, Faculty of Land and Food Systems, The University of British Columbia, Vancouver, British Columbia, Canada; Cornell University, UNITED STATES

## Abstract

Domestic rabbits (*Oryctolagus cuniculus*) are the fourth most common species admitted to the British Columbia Society for the Prevention of Cruelty to Animals (BC SPCA) shelter system. However, shelter data analysis has largely focused on cats and dogs and little is known about the population dynamics of rabbits in shelters. We analyzed five years of rabbit records (n = 1567) at the BC SPCA to identify trends in intake and predictors of length of stay (LOS) of rabbits. The majority of rabbits were surrendered by their owners (40.2%), with most rabbits being surrendered for human-related reasons (96.9%). Overall, rabbit intakes decreased over the study period. When analyzing by month of intake, rabbit intakes were found to be the highest in May. Most rabbits in our data were adults (46.7%), non-brachycephalic (66.7%), erect-eared (82.5%), short-furred (76.2%), and subsequently adopted (80.3%). The median LOS of rabbits was 29 days, highlighting the pressing need to improve their time to adoption. A linear model was constructed to identify predictors of LOS of adopted rabbits (n = 1203) and revealed that intake year, intake month, source of intake, age, cephalic type, and breed size significantly predicted time to adoption for rabbits (F(37, 1165) = 7.95, *p* < 2.2e-16, adjusted R^2^ = 0.18). These findings help characterize shelter population dynamics for rabbits, shed light on the challenges associated with unwanted rabbits, and offer a foundation for animal shelters to design programs and marketing strategies tailored to reduce LOS of rabbits with particular characteristics. Shelter rabbits represent an understudied population and our study highlights the importance of further research in companion rabbits.

## Introduction

The domestic rabbit (*Oryctolagus cuniculus*) is the fourth most prevalent companion animal admitted to the British Columbia Society for the Prevention of Cruelty to Animals (BC SPCA) in 2021, representing 2.7% of the organization’s total intake [1]. However, scholarly research has largely focused on cats and dogs [2, 3]. The problem of unwanted rabbits in animal shelters remains much less investigated than for other species [4–6].

An understanding of the population dynamics of shelter animals can help guide programs to increase their adoption and improve welfare [5, 7]. Previous studies have revealed that the majority of rabbits entering shelters were adult rabbits of various breeds, exhibiting a diverse range of physical characteristics [5, 6, 8]. Rabbits were primarily surrendered by their owners for reasons related to human factors, rather than issues with animal behaviour [4–6]. These findings can help provide insights into the scope of the problem of unwanted rabbits [5]. Furthermore, there is a common belief within the sheltering community that rabbit intakes increase in the spring due to unwanted Easter presents [5]. However, to the best of our knowledge, no study has definitively supported this anecdotal belief [5, 9]. Cook and McCobb [5] found a slight but non-significant increase in rabbit intakes in May in four animal shelters in the United States (US), and Neville and colleagues [9] reported a greater number of rabbit advertisements in summer and winter in the United Kingdom (UK).

The amount of time an animal spends in shelter, from intake to exit, is known as an animal’s length of stay (LOS; [10]). Most rabbits entering shelters are subsequently adopted; however, the median LOS for rabbits appeared to be longer than that of cats and dogs in the US and the UK [5, 6]. Increased LOS has been associated with increased risk of illness and behavioural deterioration in shelters [10–12]. Therefore, understanding the factors that influence LOS can provide valuable insights for developing programs and initiatives aimed at reducing LOS. This, in turn, can enhance animal welfare while ensuring more efficient utilization of shelter resources [10]. Many studies have been conducted to investigate factors that influence LOS for other, more popular, companion animals, such as cats (e.g., [13, 14]) and dogs (e.g., [15, 16]); however, little is known about the predictors of LOS for rabbits. Ellis and colleagues [6] found no statistically significant difference in the LOS for rabbits that were surrendered due to rabbit-related reasons compared to those surrendered for human-related reasons. Physical characteristics, such as cephalic type, ear type, fur length, and coat colour have been identified as factors influencing people’s preferences for rabbits based on an online survey of static images [17]. However, the impact of these characteristics on their LOS in the shelter remains unknown [17]. Identifying predictors for rabbits’ LOS is crucial in understanding adopters’ preferences when choosing a pet rabbit and increasing rabbit adoptions in shelters.

This study focuses on rabbits, an understudied population in animal shelters, and is the first of its kind to investigate annual and seasonal variation in rabbit intakes, as well as factors that influence their LOS in British Columbia, Canada. The primary objective of this study was to explore the characteristics of rabbit intakes and identify predictors of LOS for adopted rabbits in BC SPCA shelters in Canada. Furthermore, the data were explored to investigate annual and monthly variation in rabbit intakes. We hypothesized that year, but not month, would significantly influence rabbit intakes, and that intake year, source of intake, age, health status, coat colour, cephalic type, and ear type would play a significant role in predicting LOS.

## Methods

### Description of dataset and data cleaning

Rabbit records (n = 1665) for 36 BC SPCA shelters from January 1^st^, 2017 to December 31^st^, 2021 were obtained from ShelterBuddy [18], a database utilized by all BC SPCA shelters. Only records with a final outcome (e.g., adopted, euthanized, transferred, etc.) were included in the analysis, and rabbits with an ongoing status were excluded. Records that were out of date ranges (n = 78) were removed, as were rabbits entering the shelter for emergency boarding, evacuation, or euthanasia requests, and rabbits with an unknown final status (n = 20), resulting in a final sample size of 1567 rabbits. LOS was calculated by ShelterBuddy, excluding foster days, protective custody days, court and stray hold days from the calculation. Duplicated records were examined and represented 108 rabbits that were adopted more than once. These records were included in the analysis, with their LOS being calculated as the difference between each intake and outcome date.

The source of intake was regrouped into six categories for simplicity: humane officer, offspring, transfer in, stray, owner surrender, and return. Similarly, the outcome variable was regrouped into seven categories: adopted, euthanized, natural death, redeemed, released, escaped, and transfer out. Age was classified into maturity categories based on the life stages of rabbits. Rabbits younger than six months were defined as “young” as most rabbits reach sexual maturity by six months of age [19]. Rabbits between six months and one year old were defined as “adolescent” as marked behavioral changes are associated with reproductive changes at this stage [19]. Rabbits between one and five years old were defined as “adult”, and those older than five years were defined as “senior”. Five years was chosen as the starting point for the “senior” category based on veterinary suggestion of beginning geriatric veterinary exams at five years old [6].

The primary coat colour of rabbits, initially entered as 42 different categories by shelter staff, was regrouped into five categories: beige/brown, black, blue/grey, hairless, and white. Certain physical characteristics of rabbits, such as cephalic type (brachycephalic or non-brachycephalic), fur length (long or short), ear type (erect or lop), and breed size (converted from lbs to kg; giant: >5.4 kg; large: 4 to 5.4 kg; medium: 2 to 4 kg; small: < 2 kg), were further classified based on 39 primary breed categories entered by shelter staff and the maximum senior weight recognized by the American Rabbit Breeders Association guidelines [20]. In cases where it was not possible to determine these characteristics from the primary breed description, rabbits were classified as unknown for that specific characteristic.

Surrender reasons were classified into ten categories (abandoned, behaviour, expense, feral, housing, offspring, owner life-related, owner health-related, owner other pet-related, and other) and described in Table 1. Behaviour was assigned to rabbit-related reasons, while the remaining nine categories were considered to be human-related reasons.

**Table 1:** Surrender reasons and descriptions.

Surrender Reason	Description
Abandoned	Abandoned by a friend, relative, or tenant, found abandoned.
Behaviour	Bit a person or another animal, destructive behaviours, growling or lunging, housetraining or spraying issues, etc.
Expense	Unable to afford general or veterinary expenses.
Feral	Free-roaming on people’s properties.
Housing	Moving, unable to find or afford pet-friendly housing, landlord would not allow pets, etc.
Offspring	Pregnant animal, unwanted litters.
Owner Health-related	Allergies, injury, illness, hospitalization, etc.
Owner Life-related	No time, too much responsibility, children are not ready for pets, divorce, violence in family, new baby, travel, holidays, school, work, jail, etc.
Owner Other Pet-related	Too many other animals, aggression by other animals, etc.
Other	Constable visit, rescue, etc.

### Analyses

Data analyses were conducted in R Studio Version 4.2.2. Descriptive data for intake, physical characteristics, and surrender reasons were presented in tables as the number of individuals and their percentage. Number of rabbit intakes by year and month were presented in bar graphs. Chi-squared goodness of fit tests were used to assess annual and monthly variation in the total number of rabbit intakes.

The median and range of LOS was reported for adopted and euthanized rabbits, and only complete records of adopted rabbits (n = 1203) were included in a multiple linear regression model to identify predictors of LOS [21]. The model assessed 11 independent variables, including intake year, intake month, source of intake, age, sex, Asilomar Accords category, primary coat colour, cephalic type, ear type, fur length, and breed size.

The Asilomar Accords defines four categories, Healthy, Treatable-Rehabilitatable (TR), Treatable-Manageable (TM), and Unhealthy and Untreatable (UU), and is used as a proxy for health and behavioral status in BC SPCA shelters [22]. Healthy animals refer to those over eight weeks old with no sign of behavioral or temperamental characteristic that poses a health or safety risk and no sign of disease, injury, or congenital or hereditary condition that adversely affects or is likely to adversely affect their health [22]. The TR category includes animals likely to become healthy with medical, foster, behavioral, or other care, while the TM category includes animals unlikely to become healthy regardless of care provided but can maintain a satisfactory quality of life with medical, foster, behavioural, or other care [22]. The UU category includes those over eight weeks old with a behavioral or temperamental characteristic that poses a health or safety risk or are suffering from a disease, injury, or congenital or hereditary condition that adversely affects or is likely to adversely affect their health and are unlikely to become healthy or treatable even with care [22]. This category also includes those under eight weeks old that are unlikely to become healthy or treatable even with care [22].

A causal diagram was created to identify confounding and intervening variables. Because the distribution of LOS was found to be positively skewed, LOS was log-transformed using the formula log(*χ*+1) to meet the assumption of normality. The residual plot of the model using log LOS was visually inspected for linearity, homoscedasticity, and independence of errors. The adjusted R square, F-statistic, degree of freedom, and *p-value* were reported. Tukey’s Honestly Significant Difference (HSD) test using Bonferroni correction were performed to compare each statistically significant variable found in the model. The means, medians and distributions of the data were presented in boxplots and raincloud plots. The medians were used as a comparison measure to interpret the data as the distribution of LOS was positively skewed. A *p-value* of < 0.05 was considered statistically significant.

## Results

### Intakes

A total of 1567 rabbits (including 108 rabbits that were adopted more than once) were admitted to BC SPCA shelters from January 1^st^, 2017 to December 31^st^, 2021 (Table 2). Most rabbits entering the shelter were surrendered by their owners (40.2%), followed by stray rabbits (34.7%) and intake from humane officers (12.3%). Most rabbits (46.7%) were between one year and five years old, 21.9% were younger than six months, 21.6% were between six months and one year old, and 4.2% were older than five years. There was a similar number of male and female rabbits entering the shelters. Most rabbits were classified as healthy (45.9%) within the Asilomar Accords categories. Most rabbits had a beige/brown (34.1%) primary coat colour, with other common coat colours including white, black, and blue/grey. Most rabbits were non-brachycephalic (66.7%), erect-eared (82.5%), and short-furred (76.2%). Rabbits at the shelter varied in breed sizes. Large breeds accounted for the majority (45.3%), followed by small breeds (30.1%), medium breeds (14.1%), and giant breeds (4.8%). Lastly, most rabbits entering BC SPCA shelters were adopted (80.3%), while 9.8% were euthanized, 5.3% died naturally, and 2.7% were returned to their owner.

**Table 2:** Breakdown of admission data and physical characteristics of rabbits upon intake (n = 1567).

Category	n	% Admissions
Intake Year		
2017	439	28.0
2018	329	21.0
2019	281	17.9
2020	264	16.8
2021	254	16.2
Source of Intake		
Humane Officer	193	12.3
Offspring	76	4.9
Owner Surrender	630	40.2
Returns	103	6.6
Stray	543	34.7
Transfer In	22	1.4
Age^a^		
Less than 6 months	343	21.9
6 months to 1 year	339	21.6
1 year to 5 years	732	46.7
More than 5 years	65	4.2
Unknown	88	5.6
Sex^a^		
Male	756	48.2
Female	698	44.5
Unknown	113	7.2
Primary Coat Colour^a^		
Beige/Brown	534	34.1
Black	353	22.5
Blue/Grey	273	17.4
Hairless	4	0.3
White	403	25.7
Asilomar Accords Category^a^		
Healthy	719	45.9
Treatable-Rehabilitatable (TR)	588	37.5
Treatable-Manageable (TM)	75	4.8
Unhealthy and Untreatable (UU)	184	11.7
Unknown	1	0.1
Cephalic Type^a^		
Brachycephalic	371	23.7
Non-Brachycephalic	1045	66.7
Unknown	151	9.6
Ear Type^a^		
Erect	1293	82.5
Lop	191	12.2
Unknown	83	5.3
Fur Length^a^		
Long	232	14.8
Short	1194	76.2
Unknown	141	9.0
Breed Size^a^		
Small	472	30.1
Medium	221	14.1
Large	710	45.3
Giant	75	4.8
Unknown	89	5.7
Outcome^a^		
Adopted	1258	80.3
Euthanized	154	9.8
Escaped	1	0.1
Natural Death	83	5.3
Returned to Owner	43	2.7
Released	7	0.4
Transfer Out	21	1.3

^a^ includes duplicated individuals that were returned and adopted repeatedly

Reasons for surrender were recorded for 649 rabbits (Table 3). The majority of rabbits (96.9%) were surrendered for human-related reasons, such as the owner’s life circumstances (24.5%), housing issues (18.8%), and unwanted litters (16.9%). On the contrary, only 3.1% of rabbits were surrendered for rabbit-related behaviour reasons.

**Table 3:** Surrender reasons (n = 649).

Surrender Reason	n	% of Surrender	Total n	Total % of Surrender
**Rabbit-Related**			20	3.1
Behaviour	20	100		
**Human-Related**			629	96.9
Abandoned	11	1.7		
Expense	37	5.9		
Feral	16	2.5		
Housing	118	18.8		
Offspring	106	16.9		
Owner Health-related	61	9.7		
Owner Life-related	154	24.5		
Owner Other Pet-related	102	16.2		
Other	24	3.8		

**Fig 1:**
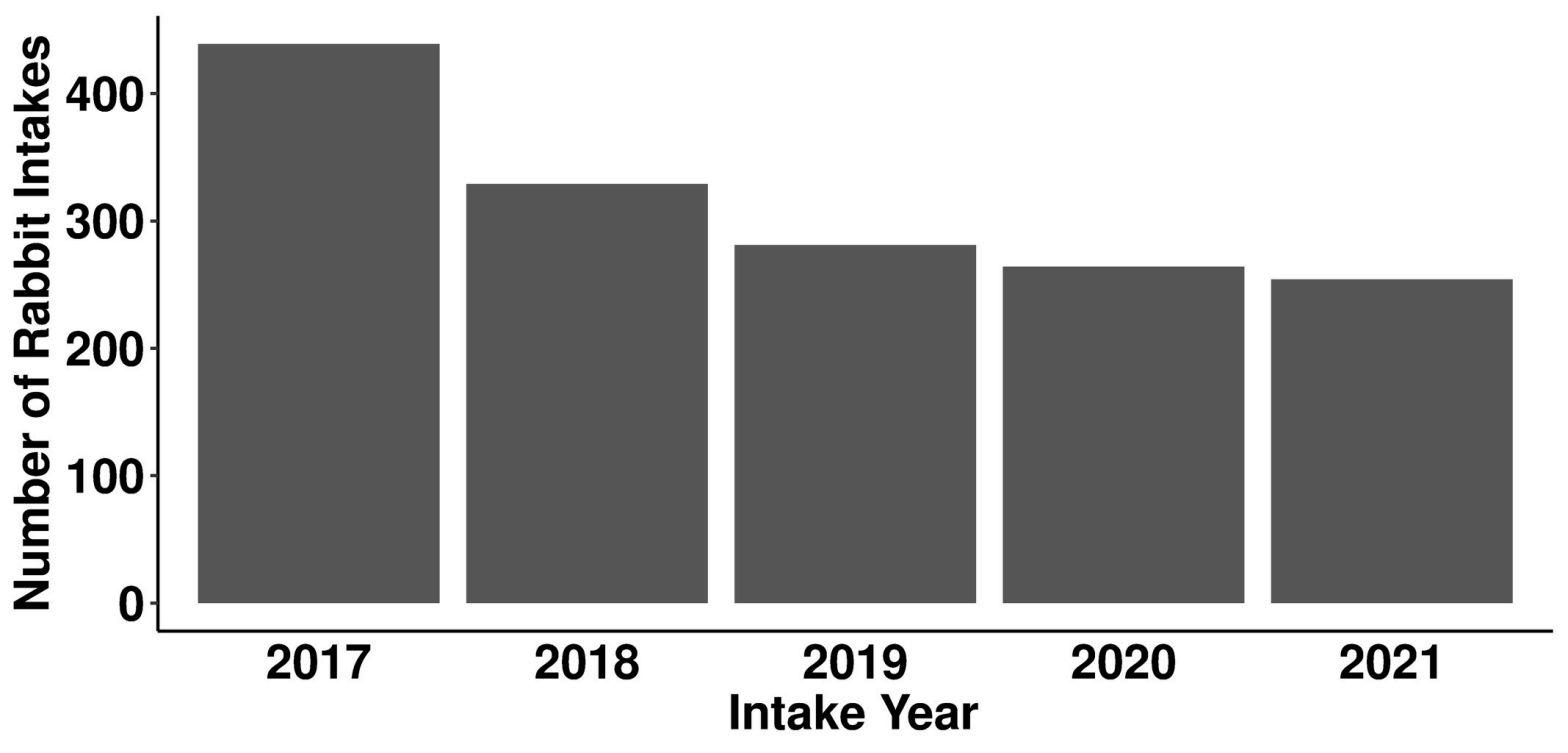
Number of rabbit intakes by intake year.

**Fig 2:**
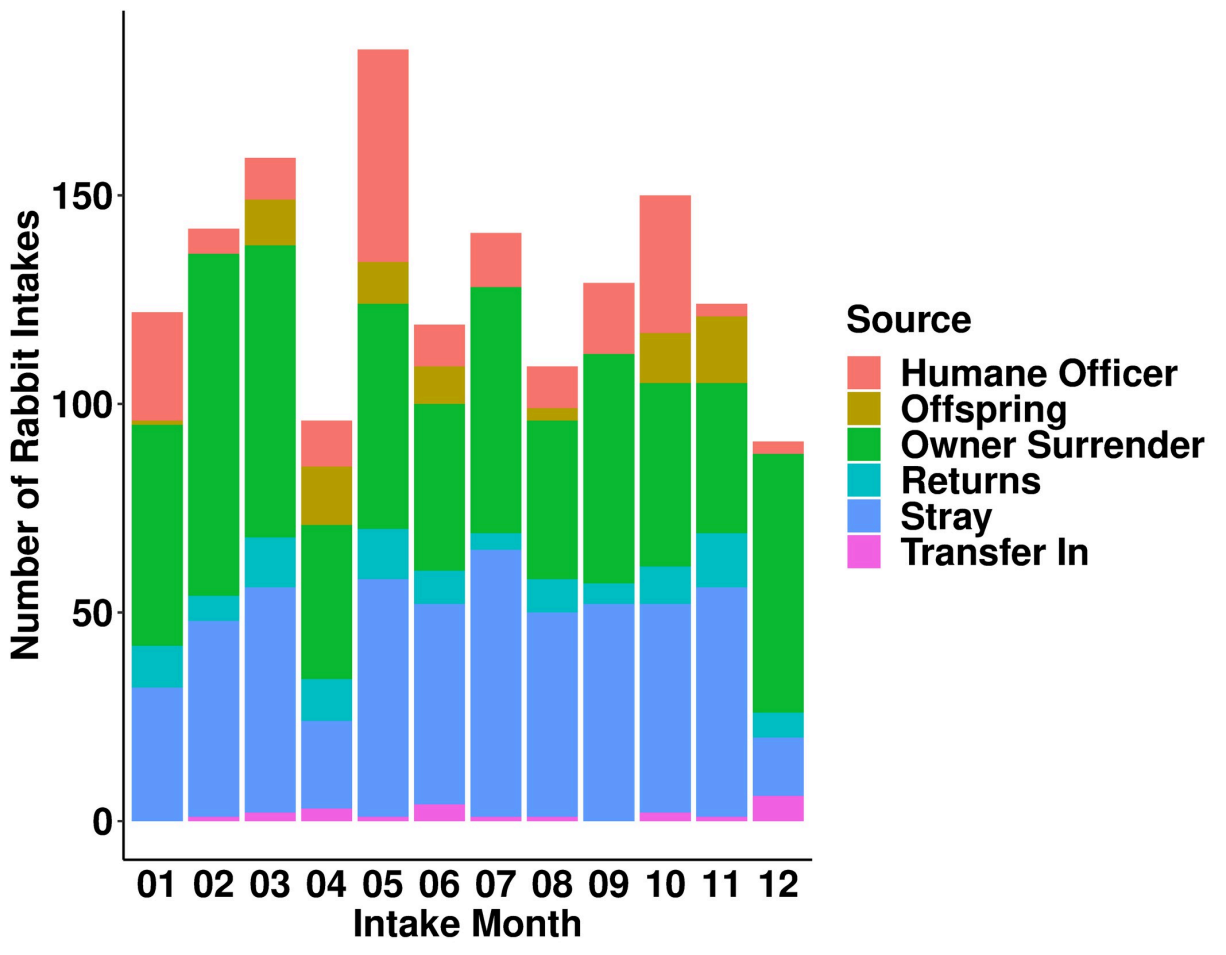
Number of rabbit intakes by intake month and source of intake. Each bar represents the number of rabbit intakes each month. The colours within each bar represent the sources of intake.

**Fig 3:**
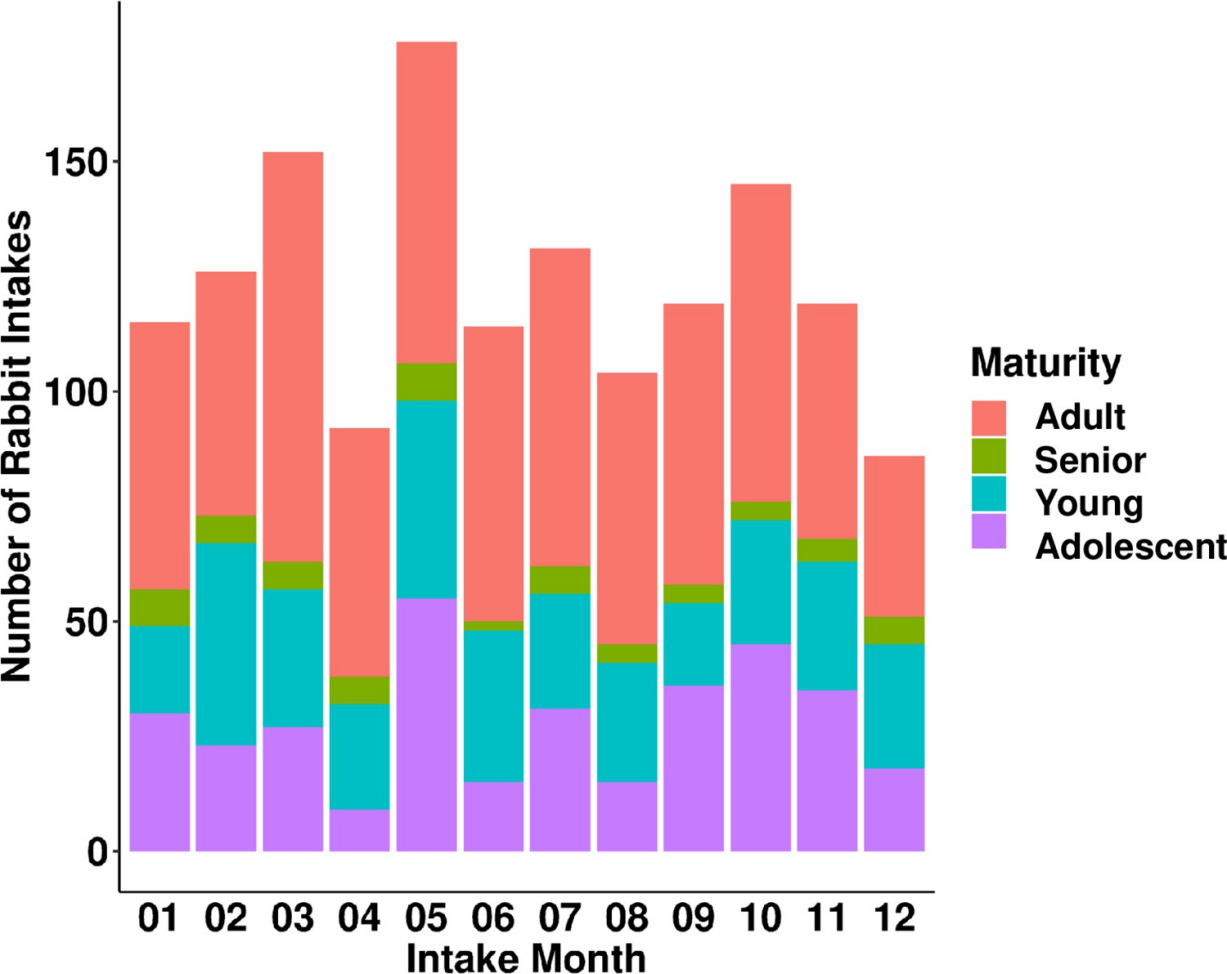
Number of rabbit intakes by intake month and age. Each bar represents the number of rabbit intakes each month. The colours within each bar represent the age categories of the rabbits.

Chi-squared test revealed that the effect of year on rabbit intakes was statistically significant (χ^2^(4) = 73.507, *p* = 4.121e-15; Fig 1). Rabbit intakes were the highest in 2017 (n = 439) and lowest in 2021 (n = 254). Month also had a significant effect on rabbit intakes (χ^2^(11) = 60.244, *p* = 8.352e-09; Figs 2 and 3). Intakes were highest in May (n = 185) and lowest in December (n = 91). Specifically, there was a higher proportion of rabbits from the humane officer source in May (28%) compared to other months (Fig 2). There was also a higher proportion of adolescent rabbits entering shelters in May (31%; Fig 3).

### Length of stay

The LOS for all adopted rabbits (n = 1258) ranged from 0 to 718 days, with a median of 29 days. The LOS of euthanized rabbits (n = 154) ranged from 0 to 275 days, with a median of 3 days.

The multiple linear regression model examining the influence of intake year, intake month, source of intake, age, sex, Asilomar Accords category, primary coat colour, cephalic type, fur length, ear type, and breed size on the LOS of adopted rabbits (n = 1203) was statistically significant (F(37, 1165) = 7.95, *p* < 2.2e-16, adjusted R^2^ = 0.18). Intake year, intake month, source of intake, age, cephalic type, and breed size, but not sex, Asilomar Accords category, primary coat colour, fur length, or ear type significantly influenced rabbits’ LOS (Table 4).

**Table 4:** Multiple linear regression model of log LOS of adopted rabbits (n = 1203).

Predictor	Median LOS	F	df	*p*
Intake Year		31.65	4	< 2e-16
2017	39			
2018	44			
2019	31			
2020	18			
2021	22			
Intake Month		2.93	11	7.8e-4
January	34			
February	27			
March	26			
April	23			
May	51			
June	23			
July	27			
August	28			
September	32			
October	40			
November	30			
December	18			
Source of Intake		4.31	5	6.8e-4
Humane Officer	47			
Offspring	58			
Owner Surrender	28			
Returns	25			
Stray	27			
Transfer In	20			
Sex		2.49	1	1.2e-1
Male	28			
Female	30			
Age		24.84	1	7.2e-7
Asilomar Accords Category		1.63	2	2.0e-1
Healthy	27			
Treatable-Rehabilitatable (TR)	41			
Treatable-Manageable (TM)	32			
Primary Coat Colour		2.24	3	8.2e-2
Beige/Brown	28			
Black	30			
Blue/Grey	24			
White	32			
Cephalic Type		20.26	2	2.3e-9
Brachycephalic	21			
Non-Brachycephalic	33			
Unknown	32			
Fur Length		2.48	2	8.4e-2
Long	25			
Short	30			
Unknown	33			
Ear Type		0.39	2	6.8e-1
Erect	30			
Lop	21			
Unknown	41			
Breed Size		7.52	4	5.5e-6
Small	25			
Medium	30			
Large	34			
Giant	14			
Unknown	27			

The median LOS in days is presented for each category of each predictor, with the exception of age which was coded as a continuous predictor. The F-value (F), degree of freedom (df), and *p-value* are presented for each predictor.

**Fig 4:**
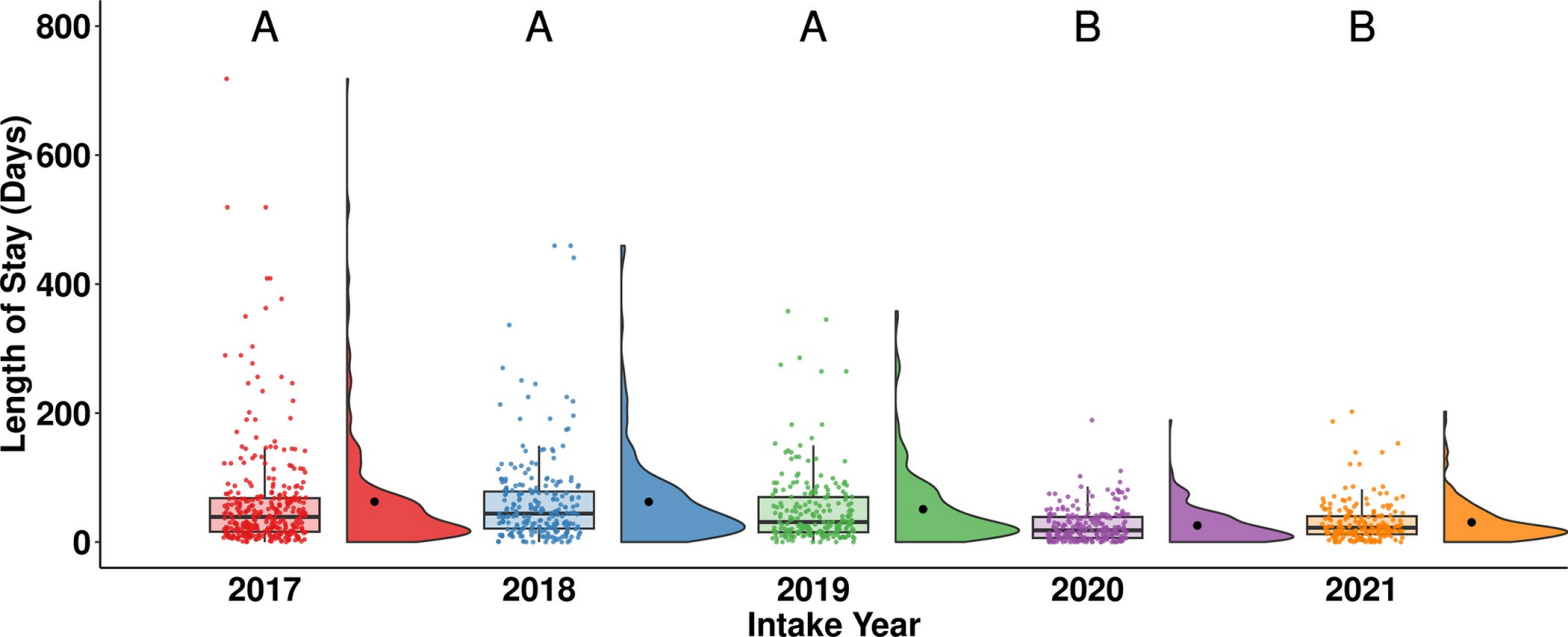
LOS of adopted rabbits by intake year. The central line of the boxplots represents the median LOS, with 25% and 75% percentiles denoted by the lower and upper bounds, the whiskers represent the range, and the dots are representing outlying values. The raincloud plots show the distribution and the mean LOS. Letters denote significant differences in LOS between intake years.

Post-hoc analyses revealed that rabbits that entered the shelter in 2020 (median = 18, n = 221) and 2021 (median = 22, n = 197) had a significantly shorter LOS than rabbits that entered the shelter in 2017 (median = 39, n = 328, *p* < 0.001), 2018 (median = 44, n = 240, *p* < 0.001), and 2019 (median = 31, n = 217, *p* < 0.001; Fig 4).

**Fig 5:**
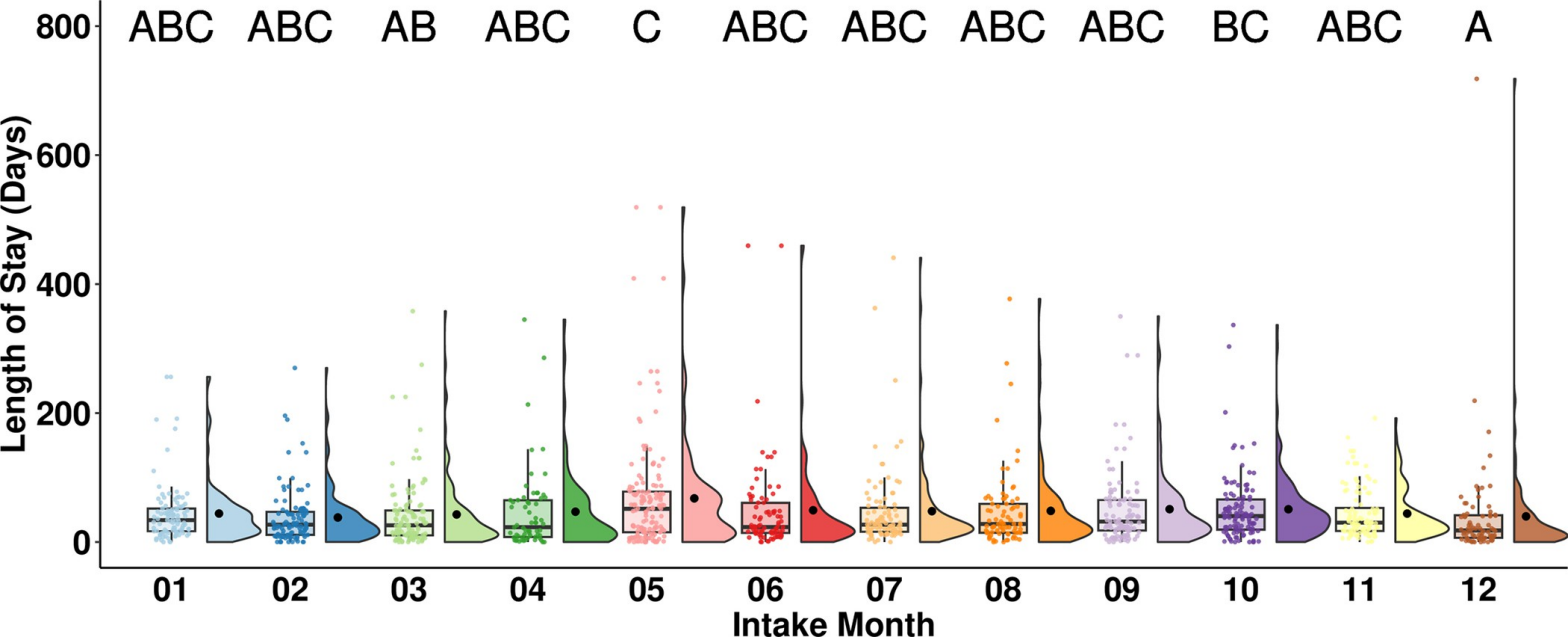
LOS of adopted rabbits by intake month. The central line of the boxplots represents the median LOS, with 25% and 75% percentiles denoted by the lower and upper bounds, the whiskers represent the range, and the dots are representing outlying values. The raincloud plots show the distribution and the mean LOS. Letters denote significant differences in LOS between intake months.

Rabbits entering the shelter in March (median = 26, n = 117) had a statistically significantly shorter LOS than rabbits entering the shelter in May (median = 51, n = 148, *p* = 0.026), and rabbits entering the shelter in December (median = 18, n = 78) had a statistically significantly shorter LOS than rabbits entering the shelter in May (median = 51, n = 148, *p* = 0.001) and October (median = 40, n = 113, *p* = 0.015; Fig 5).

**Fig 6:**
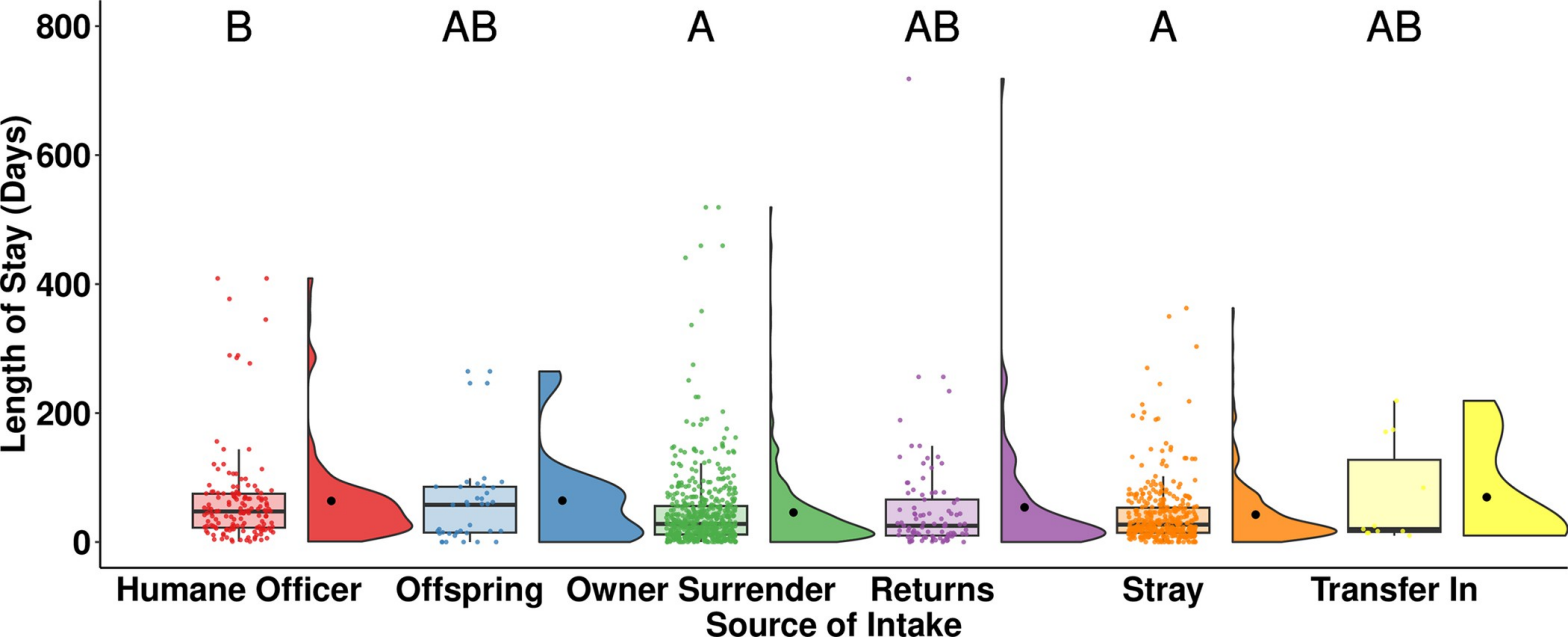
LOS of adopted rabbits by source of intake. The central line of the boxplots represents the median LOS, with 25% and 75% percentiles denoted by the lower and upper bounds, the whiskers represent the range, and the dots are representing outlying values. The raincloud plots show the distribution and the mean LOS. Letters denote significant differences in LOS between sources of intake.

Owner-surrendered (median = 28, n = 552, *p* = 0.009) and stray (median = 27, n = 364, *p* < 0.001) rabbits had a statistically significantly shorter LOS than rabbits entering the shelter through the humane officer source (median = 47, n = 146; Fig 6). The offspring category had the longest LOS (median = 58, n = 39) among all sources of intake, while rabbits that were transferred in had the shortest LOS with a median of 20 days (n = 11), but the small sample size may have precluded statistical significance.

**Fig 7:**
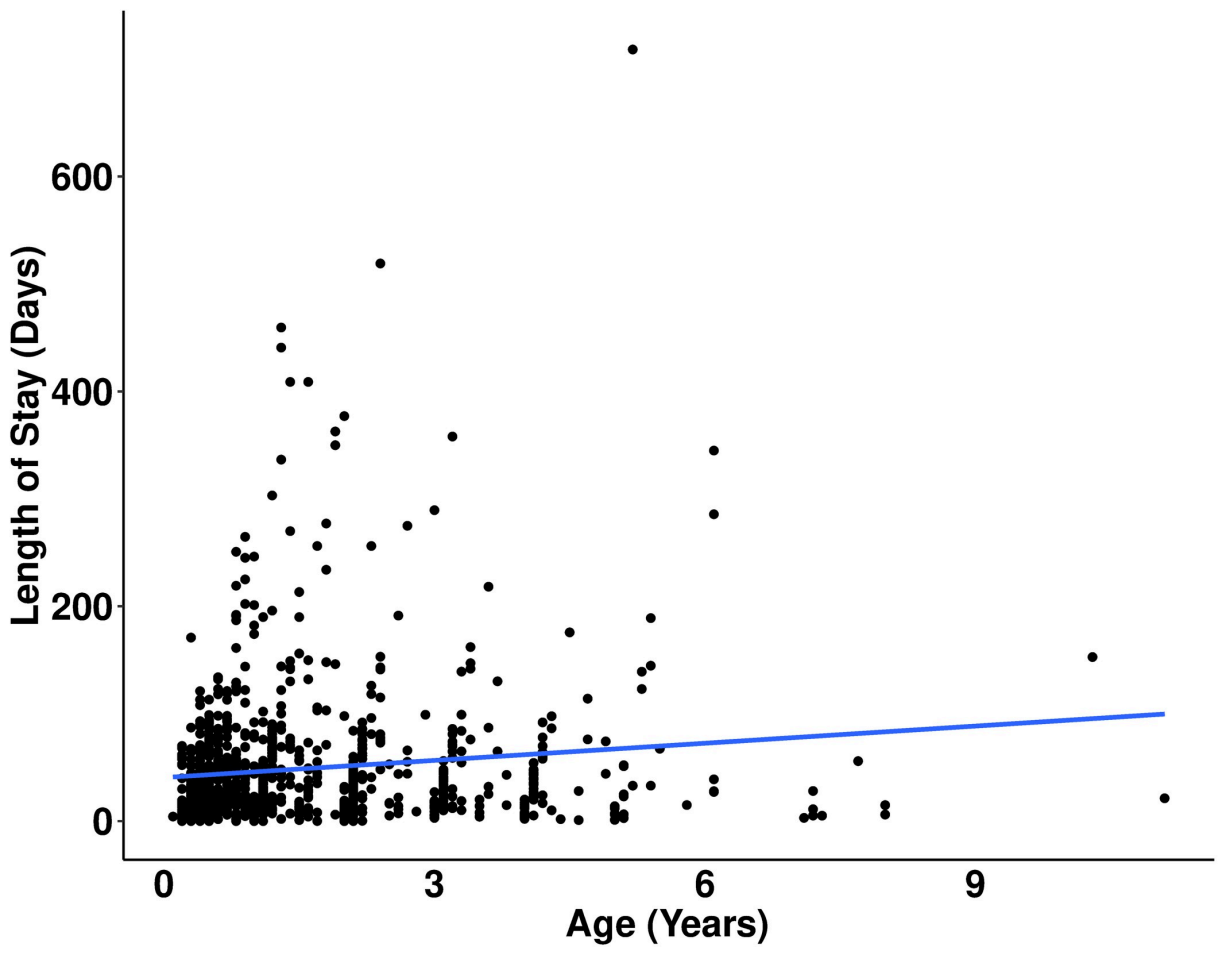
Relationship between LOS and age in adopted rabbits. LOS in days is shown on the y-axis and age in years is shown on the x-axis.

Age was found to be a statistically significant predictor of LOS, with younger rabbits experiencing a shorter LOS in shelters (Fig 7). For every one-year increase in age, the LOS increased by 14%.

**Fig 8:**
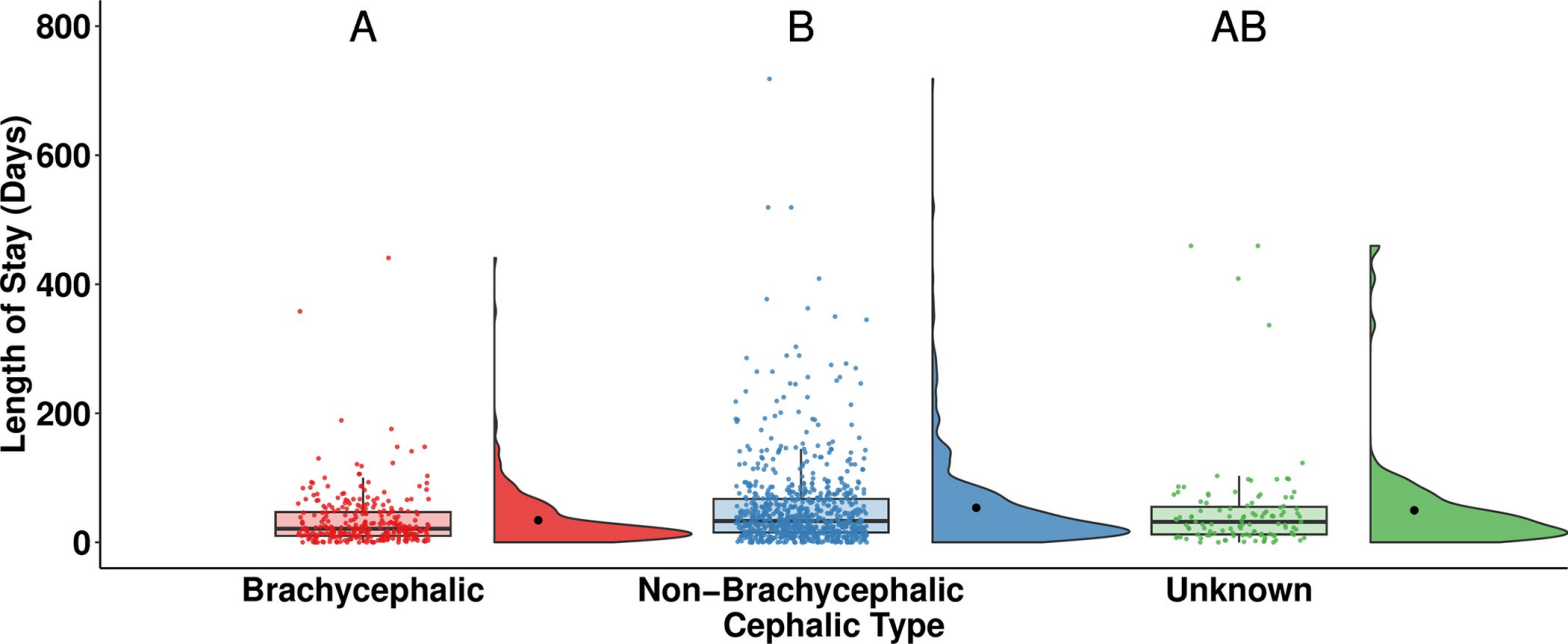
LOS of adopted rabbits by cephalic type. The central line of the boxplots represents the median LOS, with 25% and 75% percentiles denoted by the lower and upper bounds, the whiskers represent the range, and the dots are representing outlying values. The raincloud plots show the distribution and the mean LOS. Letters denote significant differences in LOS between cephalic types.

Brachycephalic rabbits (median = 21, n = 300) had a statistically significantly shorter LOS than non-brachycephalic rabbits (median = 33, n = 792, *p* < 0.001; Fig 8).

**Fig 9:**
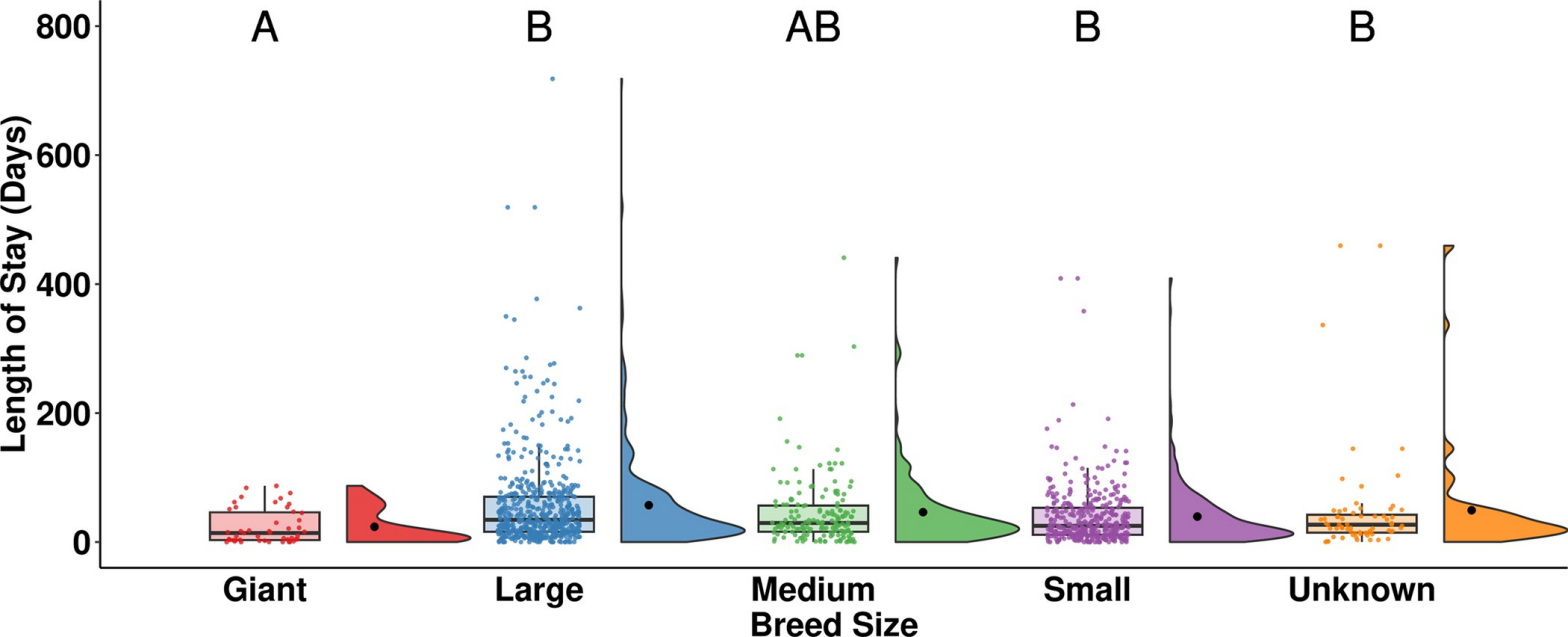
LOS of adopted rabbits by breed size. The central line of the boxplots represents the median LOS, with 25% and 75% percentiles denoted by the lower and upper bounds, the whiskers represent the range, and the dots are representing outlying values. The raincloud plots show the distribution and the mean LOS. Letters denote significant differences in LOS between breed sizes.

Giant breed rabbits (median = 14, n = 47) had a statistically significantly shorter LOS than large breed rabbits (median = 34, n = 548, *p* < 0.001), small breed rabbits (median = 25, n = 375, *p* < 0.001), and rabbits of unknown breed size (median = 27, n = 67, *p* = 0.018; Fig 9).

There was no statistically significant difference in LOS between male (median = 28, n = 620) and female rabbits (median = 30, n = 583). Asilomar Accords category also did not statistically significantly influence LOS. Rabbits assigned to a healthy (median = 27, n = 633), TR (median = 32, n = 511), or TM (median = 41, n = 59) status experienced similar LOS. No rabbits with a UU status were included in the LOS analysis due to a euthanasia outcome. LOS did not significantly differ among rabbits with primary coat colours of beige/brown (median = 28, n = 397), black (median = 30, n = 263), blue/grey (median = 24, n = 220), or white (median = 32, n = 323). There was no statistically significant difference in LOS between rabbits with long fur (median = 25, n = 193), short fur (median = 30, n = 906), and unknown fur length (median = 33, n = 104). There was no statistically significant difference between rabbits with erect ears (median = 30, n = 1002), lop ears (median = 21, n = 142), or unknown ear type (median = 41, n = 59).

## Discussion

### Intakes

A total of 1567 rabbits entered BC SPCA shelters from 2017 to 2021. The most common source of intake was from owners surrendering their rabbits (40.2%). This source represented the largest proportion of rabbit intakes in Canada (47.8%; [4]), the US (77.3%; [5]) and the UK (59.5%; [6]). The data highlight the importance of interventions focused on reducing rabbit surrenders to decrease rabbit intakes into animal shelters. Understanding the reasons for surrender can provide insights into effective interventions to reduce rabbit surrenders.

In this study, 96.9% of surrendered rabbits were given up for human-related reasons. This aligns with past findings where 87.8% to 96.6% of rabbit surrenders were attributed to human-related reasons [4–6]. Similar results have been revealed in studies on other companion animals like rats and dogs, emphasizing that human factors predominantly drive these surrenders [7, 16]. The most common human-related surrender reasons found in this study were related to the owner’s life circumstances (24.5%), housing (18.8%), and unwanted litters (16.9%). Life circumstances may include events such as divorce, the birth of a new baby, waning interest, or the lack of capability to care for rabbits. These findings indicate a potential lack of understanding among some owners about rabbit care [5, 9]. In fact, Edgar and Mullan [23] surveyed 52 pet rabbit owners in the UK and found that most respondents had limited knowledge about rabbit care needs. This highlights the crucial role that animal shelters can play in educating and supporting pet owners.

Housing-related issues remain a prevalent reason for surrenders across studies, demonstrating the need for pet-friendly housing [5, 6]. Unwanted litters have also been previously reported as a common surrender reason for rabbits, indicating the importance of spaying and neutering, broadening owner education, and enhancing accessibility to veterinary services [5, 6]. A study in the UK found that impulsive rabbit purchases were associated with a lower intention to spay or neuter, further highlighting the role of informed ownership [23]. To prevent the issue of unwanted rabbit litters, animal shelters might consider making spaying and neutering mandatory before adoption. However, accessibility to veterinarians that treat rabbits can be a barrier, and increasing the availability of rabbit spay and neuter services is important in preventing unwanted litters [5, 8].

Only 3.1% of rabbits were surrendered for animal-related reasons in this study. This is in line with previous studies on owner-surrendered rabbits, where rabbit-related reasons accounted for a small proportion of surrenders (3.4% to 12.2%; [4–6]). It has been suggested that owners might not disclose behavioural issues when surrendering animals, fearing it might affect their chances of adoption [5, 6]. This could explain the small proportion of rabbit surrenders for behavioral reasons [5, 6]. Compared to other companion animals, the percentage of rabbits surrendered for animal-related reasons is similar to that of rats (1.4%; [7]), but lower than the percentages of cats (33.2%) and dogs (46.4%) surrendered for behavioral reasons [24]. Compared to cats and dogs, rabbits and rats may be less likely to exhibit behaviours perceived as problematic or dangerous by owners, as many are generally confined and may pose a lesser risk of causing serious injuries, resulting in fewer surrenders for behavioral reasons [5, 7].

The next most common source of intake was stray rabbits (34.7%). This is similar to the proportion of stray rabbits in Canada (38.1%; [4]) and the UK (27.3%; [6]), but higher than that found in the US (15.6%; [5]). Such a difference may be explained by geographical differences and requires further research into the high proportion of stray rabbits in Canada. As stray rabbits may be escaped pets, Ellis and colleagues [6] suggested promoting microchipping to help owners locate escaped stray rabbits. Shelters might also consider microchipping all rabbits before adoption to increase the chances of reuniting escaped rabbits with their owners. Alternatively, some stray rabbits might be pets deliberately released by their owners. The abandonment of domestic rabbits outside has become a significant issue in British Columbia, leading to an increase in feral rabbit populations [25]. Our results reflect the importance of preventing rabbit abandonment and formulating strategies to manage feral rabbit populations in British Columbia. Trap-Neuter-Return (TNR) programs, proven effective for managing feral cat populations [26], could be adapted for rabbits. For example, Long Beach City College in California implemented a similar TNR program targeting feral rabbits and successfully sterilized 500 rabbits and rehomed 350 rabbits [27]. As such, TNR/adopt programs can potentially help manage the feral rabbit population. However, currently such programs are not permitted for rabbits in British Columbia due to the classification of feral rabbits as wildlife.

Most rabbits entering shelters were adults (age between one and five years; 46.7%). This is similar to the findings of Ellis and colleagues [6], where adult rabbits (age between six months and five years) represented 56% of total intake. Likewise, Díaz-Berciano and Gallego-Agundez [8] found that adult rabbits (age between six months and five years) represented 64% of total intake, while Cook and McCobb [5] found that adult rabbits (age between one and six years) represented 71.1% of intakes. A standardized classification of rabbit life stages by age would facilitate more consistent comparisons across studies.

There was a similar proportion of male (48.2%) and female (44.5%) rabbits entering BC SPCA shelters. Likewise, proportions of male and female rabbits were similar in two rehoming centers in the UK [6] and in a foster home network in Spain [8].

A large percentage (45.9%) of rabbits included in these analyses were assigned a healthy Asilomar Accords category. As only records with a final outcome were included in the analysis, it is possible that unhealthy rabbits may not have been included in the analysis if they stayed at the shelter for a very long time. Nonetheless, our finding is comparable to a study in the UK, which found that 61.5% of rabbits surrendered were healthy, while 38.5% of rabbits surrendered had health issues [6]. However, Díaz-Berciano and Gallego-Agundez [8] found a greater proportion of unhealthy rabbits (65.7%). This might be because the foster home network they studied gave priority to admitting animals considered vulnerable [8].

Physical appearance varied among rabbits in this study. The most common primary coat colour was beige/brown (34.1%), followed by white (25.7%), black (22.5%), and blue/grey (17.4%). In contrast, Ellis and colleagues [6] found that black (24%) and white (22%) were the most common coat colours, with other common colours including grey, mixed, and brown. Such variations may arise from challenges in classifying rabbit coat colours [6], as interpretations by staff can be subjective.

As for physical traits, most rabbits in the present study were non-brachycephalic (66.7%). This contrasts with findings from Gosling and colleagues [28], where the dominant rabbit breeds in the UK, like Mini Lops, Netherland Dwarfs, and Lionheads, were all brachycephalic and represented 81.8% of the population studied. Additionally, a large proportion of rabbits had erect ears (82.5%) in this study, but others have found Mini Lops to be the most commonly bred breed in the UK (63.6%; [28]) and Dwarf Lops to be the most commonly purchased breed in the UK (65%; [23]). Regarding fur length, most rabbits in the present study had short fur (76.2%). This result aligns with findings in the UK, where the most popular breeds are short-furred, except for the Lionhead breed that has longer fur [23, 28]. When considering breed size, large breed rabbits were found to be the most common (45.3%). This also differed from trends in the UK, where small to medium size breeds are more popular [28]. Mini Lops, a medium size breed, represented 63.6% of rabbits being bred [28].

Overall, differences in physical characteristics between this study and studies in the UK may be reflective of geographical differences in the popularity of brachycephalic, lop-eared, and small to medium sized rabbits [23, 28]. It is also important to note that while this study focused on rabbits in animal shelters, studies in the UK focused on rabbits being bred or purchased [23, 28].

The majority of rabbits in this study were adopted (80.3%), with 9.8% euthanized. Compared to a study from the US [5], there was a higher adoption rate and a lower euthanasia rate for rabbits. This difference could be influenced by shelter policies, as the BC SPCA does not euthanize animals with a healthy Asilomar Accords category. The adoption rate for rabbits is lower than that for dogs at the BC SPCA (92.4%; [16]), but higher than that for rats at the BC SPCA (59.6%; [7]). Promotions focusing on the adoption of small mammals, such as rabbits and rats, may be beneficial for increasing their adoption rates. The euthanasia rate for rabbits in this study is comparable to that for dogs at the BC SPCA (7.6%; [16]), but much lower than that for rats at the BC SPCA (32.1%; [7]). The higher euthanasia rate for rats can be attributed to the policy of assigning most neonatal rats as UU under the Asilomar Accords category to prevent overpopulation [7].

Intake year significantly influenced the number of rabbit intakes, with the number of intakes decreasing each year (Fig 1). Intakes in 2017 represented 28% of total intakes in the five-year study period, while intakes in 2021 represented only 16%. One possible explanation is the emergence of rabbit haemorrhagic disease (RHD), a highly contagious and fatal disease that was found in North America from 2018 to 2022 [29]. Animal shelters that were not obligated to accept domestic rabbits may have declined new intakes to minimize disease spread in shelter and to protect resident rabbits, resulting in a decrease in rabbit intakes. Furthermore, it has been reported in Canada and the US that the COVID-19 pandemic may have decreased overall animal intakes [3, 30, 31].

The month of intake had a significant impact on the number of rabbit intakes (Figs 2 and 3). In this study, rabbit intakes were highest in May and lowest in December. These findings differ from previous studies that investigated monthly trends in rabbit intakes [5, 6]. For example, Cook and McCobb [5] found that rabbit intakes in two US shelters peaked in July and October, with the fewest intakes in April, June, and November. In contrast, Ellis and colleagues [6] reported high intakes in January, April, and November at two rehoming centers in the UK. Such differences in monthly intake could be affected by cultural and geographical variations or the specific time period studied. In particular, the data from Ellis and colleagues [6] covered only a single year.

Our results partially support the anecdotal belief that rabbit intakes increase in the spring following Easter, as May was the month with the highest number of rabbit intakes. Cook and McCobb [5] found a slight but non-significant increase in rabbit intakes during May across four shelters in the US. However, whether these increases in May are due to unwanted Easter rabbits requires further analysis. A further analysis into the high rabbit intake for May, considering both intake source and age, revealed a noticeable influx of rabbits sourced by humane officers and adolescent rabbits (Figs 2 and 3). A plausible explanation is that owners might release unwanted rabbits outside post-Easter, leading humane officers to intervene and collect these animals. It is important to note that adolescent rabbits exhibit undesirable behavioral changes, such as spraying, aggression, and territorial behaviours [19]. Such behaviours might drive owners to abandon these animals, especially if they had made impulsive decisions to purchase or adopt without adequate knowledge about rabbit care. However, this explanation is difficult to verify, as we do not know whether the rabbits collected by humane officers had prior owners.

### Length of stay

The median LOS for adopted rabbits in this study was 29 days. The distribution of LOS was right-skewed, indicating that many rabbits had a long LOS in shelter (Max: 718 days). The median LOS in this study is comparable to the median LOS for rabbits from 24 to 34 days in four shelters in the US [5], but lower than the median LOS of 60 days in two rehoming centers in the UK [6]. The maximum LOS in this study is higher than the maximum LOS for rabbits reported in both the US (635 days; [5]) and the UK (288 days; [6]). This may be reflective of different shelter policies, as the BC SPCA does not euthanize animals with a healthy Asilomar Accords category due to a long LOS at the shelter.

The median LOS of rabbits in this study (29 days) is similar to the median LOS of 26 days for adopted rats in BC SPCA shelters [7], but comparably higher than the average LOS for adult dogs (9 days), puppies (5 days), adult cats (11 days), and kittens (8 days) reported by the BC SPCA in 2020 [32]. This suggests that small mammals like rabbits and rats tend to stay longer in shelters than cats and dogs in British Columbia. This trend has also been observed in the US and the UK, where rabbits generally take longer to be adopted than cats and dogs [5, 6]. Differences in LOS may reflect the lower popularity of rabbits as pets in comparison to cats and dogs [33]. Additionally, potential adopters might prefer other sources, such as pet stores, over animal shelters [6]. Enhancing marketing strategies and promotions focused on small mammals could be an effective strategy in diverting potential adopters from purchasing rabbits from pet stores and increasing rabbit adoptions in animal shelters.

The linear model revealed that intake year, intake month, source of intake, age, cephalic type, and breed size, but not sex, Asilomar Accords category, primary coat colour, fur length, nor ear type significantly influenced the LOS of adopted rabbits (Table 4). As only complete records of adopted rabbits were included in the model, this analysis did not take into account rabbits that were still waiting for adoption or rabbits that might not get adopted and results should be interpreted accordingly.

Rabbits that entered the shelter in 2020 and 2021 experienced a shorter LOS than rabbits that entered the shelter in 2017, 2018, and 2019 (Fig 4). The reduced LOS in 2020 and 2021 might be attributed to the impact of the COVID-19 pandemic on public interest in animal adoptions [31, 34]. For example, Morgan and colleagues [34] surveyed a national pet adoption website in Isarel and found an increased interest in dog adoptions and adoption rate and a decrease in LOS as social isolation measures tightened during the pandemic. Similar increases in adoptions and foster applications were also reported across animal shelters in the US [31]. Humane Canada [3] highlighted a historically low median LOS for cats and dogs, largely due to an increase in animals placed in foster care. These findings add to a growing body of evidence documenting the effect of the COVID-19 pandemic on animal adoptions and suggest that this impact is not limited to cats and dogs. Furthermore, fostering programs for rabbits could be effective in decreasing their LOS.

Rabbits entering the shelter in March experienced a shorter LOS than rabbits entering the shelter in May, and rabbits entering the shelter in December experienced a shorter LOS than rabbits entering the shelter in May and October (Fig 5). One possible explanation for the shorter LOS in March and December is an increase in holiday adoptions due to Easter and Christmas. In the field of animal welfare, policies that prevent animals from being adopted as gifts are highly prevalent [35]. However, a growing body of literature suggests that the risk of relinquishment does not increase for cats and dogs when they are received as gifts [35, 36]. Therefore, promoting adoptions during holidays might aid in reducing the LOS of rabbits. However, more research is required to clarify if rabbits received as gifts face a higher chance of being relinquished.

Owner-surrendered and stray rabbits had a significantly shorter LOS compared to rabbits brought into the shelter from humane officers (Fig 6). This is consistent with the findings of Hou and Protopopova [7] on adopted rats, where rats sourced by humane officers experienced a longer LOS than rats from other sources of intake. The authors suggested that animals from the humane officer source may have come from neglectful environments and spent more time receiving medical attention or behaviour modification [7]. Degree of socialization and interactions with potential adopters have been found to influence LOS and adoptions in cats and dogs [37, 38]. The mean LOS for interactive cats was approximately three times shorter than unapproachable cats in 31 shelters in the US [37]. Dogs that laid in proximity to potential adopters were more likely to be adopted, while dogs that ignored play initiation were less likely to be adopted [38]. Rabbits entering from the humane officer source may exhibit less positive and social behaviours than rabbits from other sources of intake due to their past experiences. Therefore, the development and implementation of behaviour modification programs for these rabbits might prove beneficial in reducing their LOS.

Younger rabbits experienced a shorter LOS compared to older rabbits (Fig 7). This is consistent with studies conducted in cats [13, 37], dogs [15, 16], and rats [7], where younger animals typically have shorter LOS. To reduce the LOS for mature or older animals, shelters might explore adoption campaigns tailored to them, like the “Adopt a Senior Pet Month” initiative [39].

LOS was significantly shorter for brachycephalic rabbits compared to non-brachycephalic rabbits (Fig 8). Similarly, a survey by Harvey and colleagues [17] revealed a global preference for brachycephalic rabbits over non-brachycephalic rabbits. Skull morphology has also been found to affect people’s preferences for cats and dogs [40, 41]. Mesocephalic cats were found to be the most preferred in the study by Farnworth and colleagues [40], while the popularity of brachycephalic dogs has been found to increase in Australia [41]. These preferences have significant health and welfare implications, because brachycephaly is associated with health issues in companion animals [17, 42, 43]. While much of the research has centered on brachycephalic cats and dogs, brachycephalic rabbits also face health challenges, including dental problems, tear duct infections, and respiratory issues [17, 44, 45]. However, the breeding of brachycephalic rabbits remains common, with brachycephalic breeds among the most popular in the UK [28].

Interestingly, veterinary and animal care professionals seem less inclined to prefer brachycephalic cats and rabbits, likely due to their awareness of associated health problems these animals experience [17, 40]. This suggests that public education on brachycephaly-associated health issues can influence people’s decisions in choosing a pet rabbit and help prospective adopters make informed decisions when adopting a rabbit. However, further research on the health issues of brachycephalic rabbits is required to formulate effective public education.

Breed size significantly influenced the LOS of rabbits, with giant breeds experiencing the shortest LOS (Fig 9). Similar trends have been found in cats, with rare breeds such as exotic cats experiencing a shorter LOS than more common breeds such as Domestic Shorthair [13, 14]. Giant breed dogs have also been found to experience shorter LOS than dogs of other sizes or other breed groupings [15, 46]. It has been suggested in the literature that animals of unique breeds are easily recognizable with unique features, making them potentially more desirable to adopters [15]. Our results suggest that in addition to cats and dogs, this human preference for uniqueness may be applied to rabbits as well.

Male and female rabbits experienced a similar LOS in the present study. Previous research on other companion animals has found mixed results regarding the effect of sex on LOS [14, 15, 46, 47]. Rabbits assigned to healthy, TR, or TM Asilomar Accords category experienced a similar LOS. This contrasts with previous research indicating that that TR and TM rats, as well as injured dogs and cats, were less likely to be adopted than their healthy counterparts [7, 48]. LOS did not significantly differ among primary coat colour categories in this study. While Brown and colleagues [15] found no significant influence of coat colour on the LOS of dogs, coat colour has been reported to affect LOS in other studies on dogs [16], cats [13, 47], and rats [7]. These results indicate that predictors of LOS in other companion animals may not generalize to rabbits.

Ear type and fur length did not statistically influence the LOS of rabbits in this study. However, both ear type and fur length have been found to affect people’s perceptions of rabbits [17, 49]. González-Redondo and Contreras-Chacón [49] found that university students in Spain preferred pet rabbit breeds such as Lop Dwarf, Angora, and Lionhead, which have characteristic features like lop ears and long fur, over traditional meat rabbit breeds like New Zealand White, which has erect ears and short fur. In contrast, Harvey and colleagues [17] revealed that rabbits with erect ears and short fur were preferred globally over rabbits with lop ears and long fur. The differences between these results may reflect variations in study design or cultural variations and suggest that further research is needed to understand how the physical characteristics of rabbits influence people’s preferences and their LOS in shelters [17, 49].

## Conclusions

Five years of rabbit records (n = 1567) at the BC SPCA were analyzed to identify trends in intake and predictors of length of stay of rabbits. The majority of rabbits were surrendered by their owners, with most rabbits being surrendered for human-related reasons. Most rabbits entering the shelters and included in these analyses were adults, non-brachycephalic, erect-eared, short-furred, and subsequently adopted. Overall, rabbit intakes decreased over the study period. When analyzing by month of intake, rabbit intakes were the highest in May, with a higher proportion of rabbits sourced from humane officers and adolescent rabbits, providing some evidence for the release and abandonment of unwanted domestic rabbits by owners possibly due to undesirable behavioral changes in sexually maturing adolescent rabbits. These findings help characterize shelter population dynamics for rabbits, shedding light on the challenges associated with unwanted rabbits.

Significant predictors for a rabbit’s LOS included intake year, intake month, source of intake, age, cephalic type, and breed size. These findings offer a foundation for animal shelters to design programs and marketing strategies tailored to reduce LOS of rabbits with particular characteristics. Additional research is needed to explore effective interventions that help decrease rabbit abandonment and surrenders. Future studies could explore the dynamics of adopter-rabbit interactions and owner-rabbit relationships, offering better insights into adoption drivers and surrender reasons. This could further decrease the rabbit population in animal shelters and enhance their overall welfare.

## Supporting information

S1 File Rabbit intake data. (XLSX)

S2 File Rabbit length of stay data. (XLSX)

S3 File R script. (R)
